# The effects of anastomoses between anterior and posterior circulation on postoperative prognosis of patients with moyamoya disease

**DOI:** 10.1007/s10072-024-07346-6

**Published:** 2024-01-29

**Authors:** Yuan Yuan, Xuchao He, Yin Li, Lingji Jin, Yuhan Zhu, Gaojun Lin, Libin Hu, Hang Zhou, Yang Cao, Junwen Hu, Gao Chen, Lin Wang

**Affiliations:** 1 Department of Nursing, the Second Affiliated Hospital, Zhejiang University School of Medicine, Hangzhou, China; 2 Department of Neurosurgery, the Second Affiliated Hospital, Zhejiang University School of Medicine, 88Th Jiefang Road, Hangzhou, 310009 China; 3 Clinical Research Center for Neurological Diseases of Zhejiang Province, Hangzhou, China; 4 Department of Neurosurgery, Wenling First People Hospital (The Affiliated Wenling Hospital of Wenzhou Medical University), Taizhou, China; 5 Department of Neurosurgery, School of Medicine, Hangzhou First People Hospital, Zhejiang University, Hangzhou, China

**Keywords:** Moyamoya disease, Collateral anastomoses, Early complication, Prognosis

## Abstract

**Background:**

Moyamoya disease (MMD) is a chronic ischemic cerebrovascular disease. Collateral circulation in MMD has emerged as a research focus. Our aims were to assess the impact of anastomoses between the anterior and posterior circulations on the prognosis of MMD patients.

**Methods:**

We reviewed the preoperative digital subtraction angiography images of patients with MMD who underwent revascularization surgery at our hospital between March 2014 and May 2020 and divided the patients into two groups: those with anastomoses (PtoA group) and those without anastomoses (non-PtoA group). The differences in follow-up (more than 6 months) collateral vessel establishment (Matsushima grade) and the modified Rankin Scale (mRS) were compared between the two groups as well as between the patients with different degrees of anastomoses. The early complications following revascularization were also compared between the two groups.

**Results:**

This study included 104 patients with MMD, of which 38 were non-PtoA and 66 were PtoA. There were no significant differences in Matsushima score (*P* = 0.252) and mRS score (*P* = 0.066) between the two groups. In addition, Matsushima score (*P* = 0.243) and mRS score (*P* = 0.360) did not differ significantly between patients with different degrees of anastomoses. However, the non-PtoA group had a significantly higher rate of cerebral hyperperfusion syndrome (CHS) than the PtoA group (34.2% *vs* 16.7%, *P* = 0.041).

**Conclusion:**

MMD patients without anastomoses between anterior and posterior circulations preoperatively should be vigilant of the occurrence of CHS in the early stages after revascularization.

## Introduction

Moyamoya disease (MMD) is a chronic cerebrovascular disorder characterized by stenosis or occlusion of the terminal internal carotid artery, as well as the beginning of the anterior cerebral artery (ACA) and middle cerebral artery (MCA), accompanied by the development of smog-like vessels at the skull base [1, 2]. Despite extensive research, the natural history and underlying pathophysiology of MMD remain poorly understood [3]. Moreover, MMD patients present with a diverse clinical presentation, which further complicates their management [4]. Thus, a better understanding of the factors that influence the prognosis of MMD is essential to improving patient outcomes and enhancing their quality of life.

The cerebral collateral circulation system represents a compensatory mechanism of the cerebral vasculature, functioning as an auxiliary pathway to maintain normal cerebral blood flow in cases of compromised blood supply to the major intracranial arteries [5]. As a progressive ischemic disease, MMD triggers the establishment of collateral circulation following cerebral ischemia. However, not all MMD patients will be able to develop an effective collateral circulation. The Suzuki stage has been widely used for assessing the progression of MMD as well as to some extent for assessing collateral circulation development [4, 6]. Nevertheless, given that patients with the same Suzuki stage may exhibit clinical manifestations of varying severity, some researchers consider that the predictive value of Suzuki stage in the clinical progression and prognosis of MMD patients is limited [7].

Researchers have been increasingly interested in the collateral circulation system of MMD in recent years [4, 8]. For instance, Funaki et al. [9] reported that choroidal anastomoses are closely linked to the occurrence of posterior hemorrhage in MMD and may be a key vessel responsible for this complication. This finding was subsequently confirmed by Wu et al. [10] using high-resolution magnetic resonance imaging. Therefore, investigating different collateral anastomoses may offer novel insights for the clinical diagnosis and management of MMD.

MMD often involves anastomoses between the ACA and the posterior cerebral artery (PCA), which Bonasia et al. [8] found to be present in more than half of the cerebral hemispheres. In addition, previous research has shown that the state of collateral circulation is closely related to the clinical outcome of patients with acute ischemic stroke [11, 12]. Therefore, in this study, we not only investigated the correlation between the presence of the ACA-PCA anastomoses and the clinical outcome of MMD patients but also paid attention to the effect on the collateral vessel establishment following revascularization.

## Methods

### Study design and cohort

**Fig. 1:**
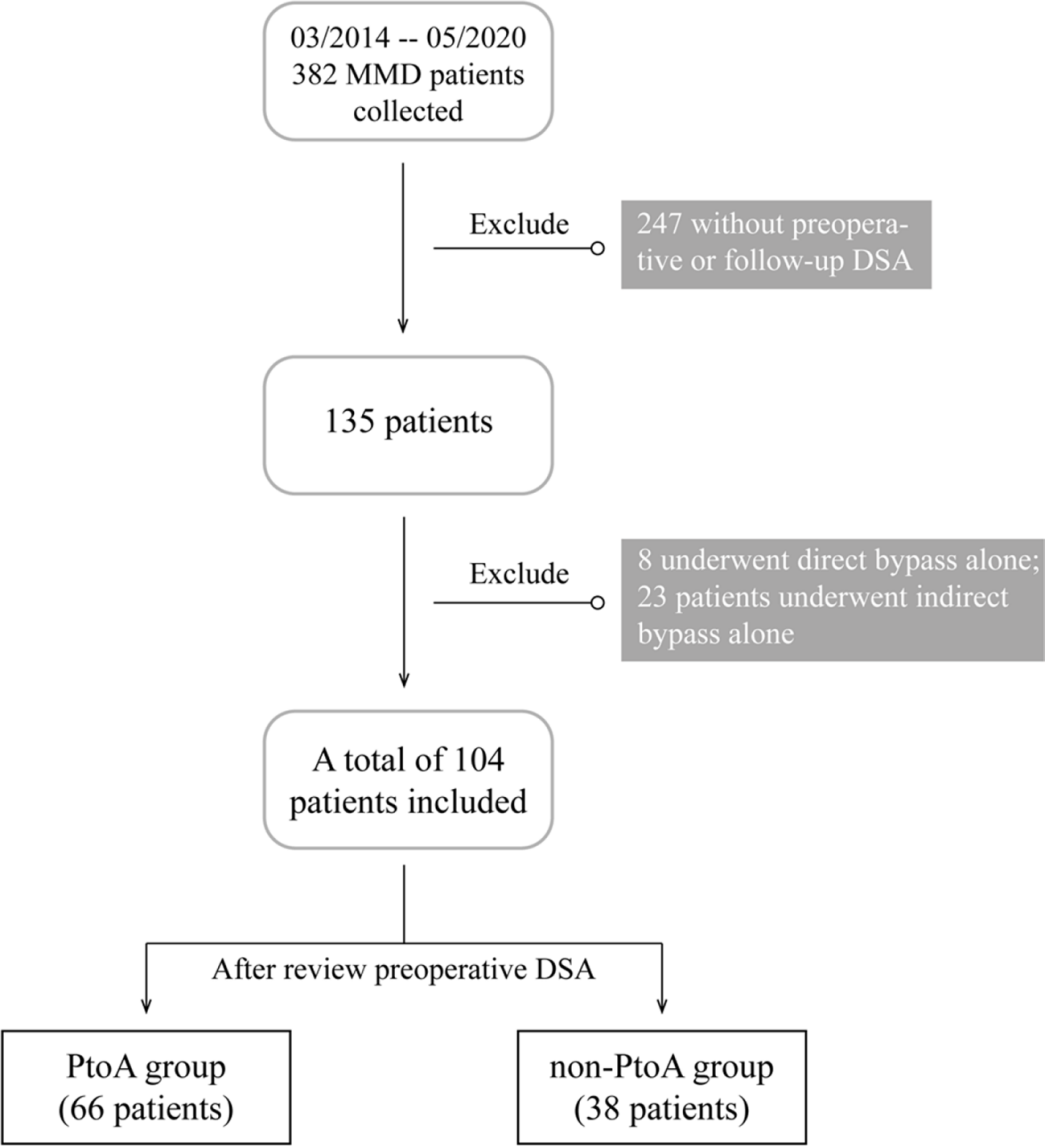
Patient flow chart of the study

We conducted a retrospective analysis of patients admitted to our hospital between March 2014 and May 2020 who were diagnosed with MMD and received revascularization. The diagnostic criteria were in line with the consensus [13]. Our study included patients over the age of 18 years who had received combined revascularization, which included both superficial temporal artery-middle cerebral artery anastomosis and encephalomyosynangiosis (EMS), and had been followed for more than 6 months. Patients who did not receive preoperative digital subtraction angiography (DSA) or who did not have DSA re-examination records during follow-up at our institution or who were treated only with direct or indirect bypass surgery were excluded from our study (Fig. 1). This study was approved by the Human Research Ethics Committee of the Second Affiliated Hospital of Zhejiang University (ID: 2020–064). The study had been carried out in accordance with the Declaration of Helsinki.

We collected general clinical information about the patients, including their age, gender, body mass index (BMI), past medical history (such as smoking, alcohol, hypertension, diabetes), type of onset (cerebral hemorrhage, ischemia, and others), duration of the onset, Suzuki stage, and follow-up interval.

### Imaging collection and assessment

We reviewed the preoperative and follow-up DSA images of all the included patients, including both the anteroposterior and lateral images of bilateral carotid angiography and vertebral angiography. Two experienced neurosurgeons with expertise in cerebrovascular disease evaluated all images jointly, and if there were any disagreements, a third neuroradiologist was consulted. Patients with more than 50% stenosis or occlusion in posterior cerebral arteries were defined as PCA involvement.

### Anastomoses between the anterior and posterior circulations

**Fig. 2:**
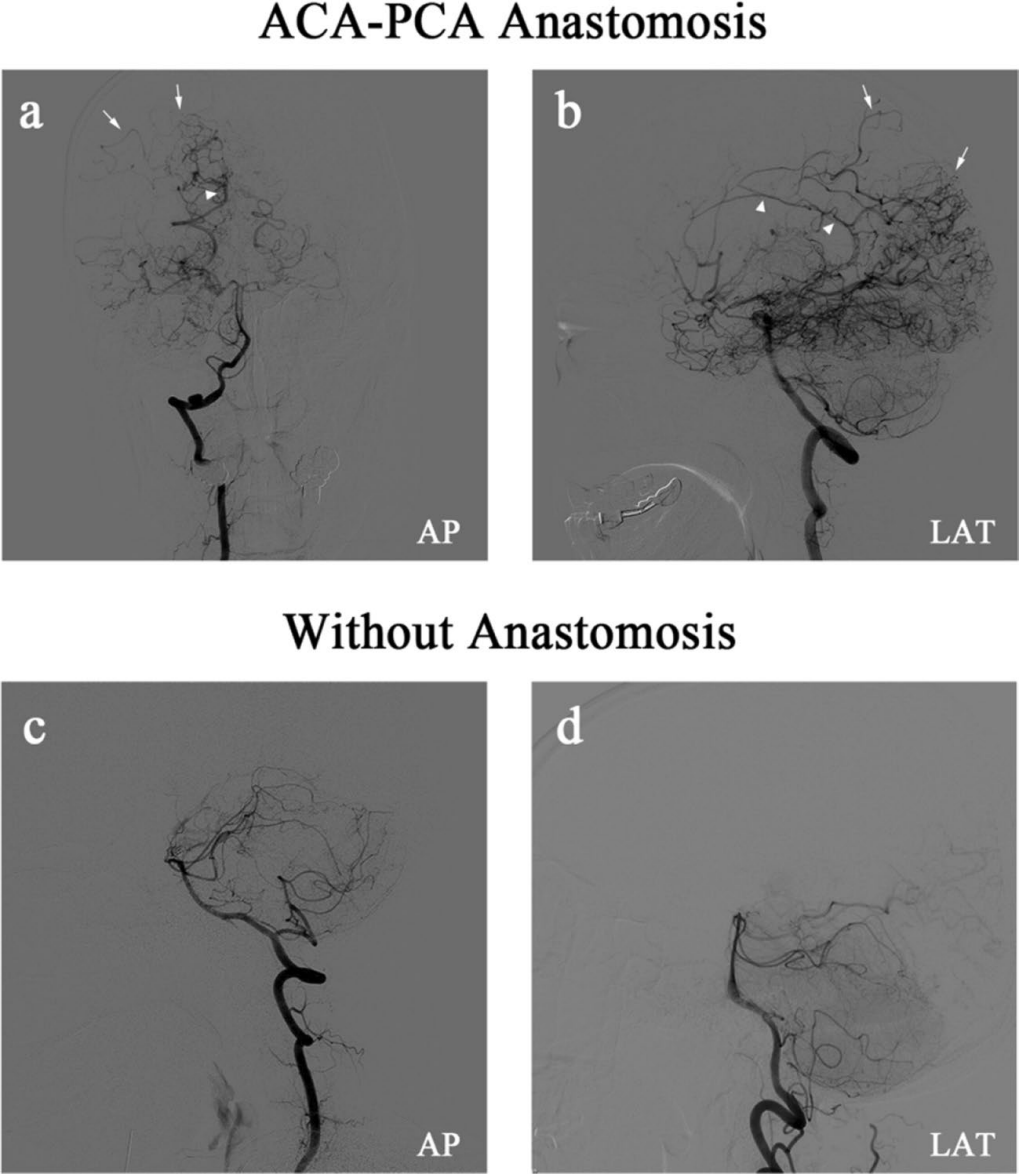
Both (**a**) and (**b**) illustrate two types of collateral from the PCA to the ACA territory through the anteroposterior (AP) and lateral (LAT) views of vertebral angiography, respectively. The triangles indicate blood flow from the PCA territory to the ACA territory via the posterior pericallosal artery. The arrowheads show the leptomeningeal connections between the PCA and the ACA territory. As can be seen in (**c**) and (**d**), patients without ACA-PCA anastomoses do not exhibit any of the above collateral patterns on their vertebral angiography

There are two types of ACA-PCA anastomoses in this study. In the first instance, the posterior pericallosal artery serves as a pathway for blood flow from the PCA to the ACA territory. The posterior pericallosal artery arises from the PCA or its branches, which can anastomose with the anterior pericallosal artery, a branch of the ACA, to fill it retrogradely. The second type is the leptomeningeal connections between the PCA and the ACA territory. These are collaterals between cortical branches of the PCA, such as the parieto-occipital artery, and terminal branches of the ACA (Fig. 2a, b), which extend to the cortical border zone. Patients displaying any of these collateral patterns on their vertebral angiography on the operation side are considered to have an ACA-PCA anastomoses, while those without an ACA-PCA anastomoses do not exhibit any collateral patterns (Fig. 2c, d). Afterwards, patients were divided into two groups according to whether they had ACA-PCA anastomoses (PtoA group) or not (non-PtoA group).

### Grading of ACA-PCA anastomoses

**Fig. 3:**
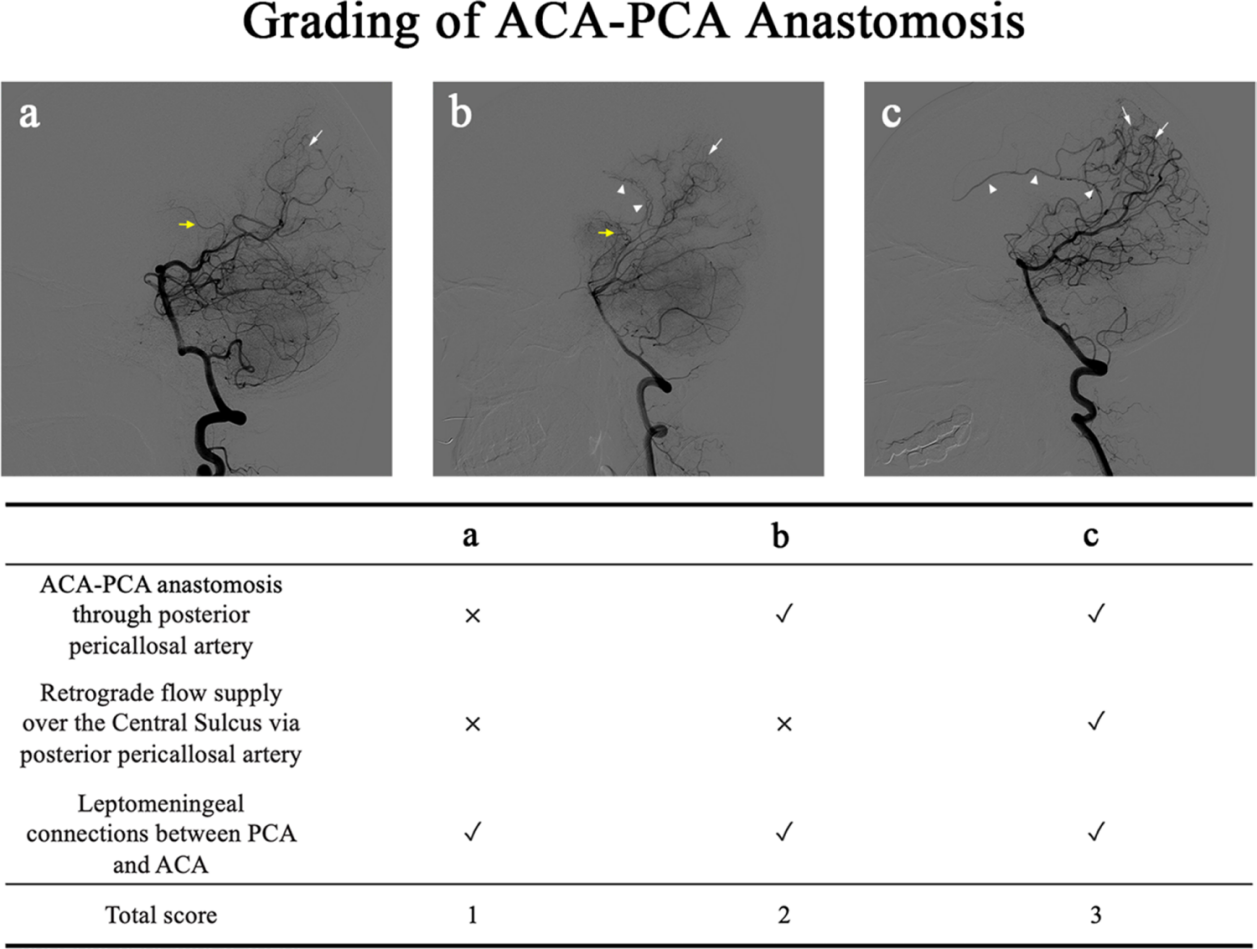
Different types and degrees of anastomoses between the ACA and the PCA. **a** Presences leptomeningeal connections from the PCA to the ACA territory, which was given a score of 1. **b** Two types of collateral from the PCA to the ACA territory, but the retrograde flow from the posterior pericallosal artery does not exceed the central sulcus. **c** Given a score of 3 due to the presence of two types of collateral and the retrograde flow supply over the central sulcus via the posterior pericallosal artery

Angiographical collateral grades were evaluated based on the types and degrees of anastomoses between the ACA and the PCA. The grading criteria referred to the results published by Liu et al. [4] as follows, and the score ranged from 0 to 3. A score of 1 was assigned to the ACA-PCA anastomoses originating from the posterior pericallosal artery, while a score of 2 was assigned to the retrograde flow supply over the central sulcus via the posterior pericallosal artery. In addition, the presence of leptomeningeal connections from the PCA to the ACA territory was also given a score of 1. Thus, the lowest score is 0 points, which indicates that the patient does not have any ACA-PCA anastomoses, and the highest score is 3 (Fig. 3).

### Assessment of collateral vessel establishment following revascularization

Collateral vessel establishment was evaluated according to the Matsushima grade [14] by observing the lateral views of the follow-up DSA images. The new development of collateral circulation through the bypass was classified into three degrees according to the extent of the blood supply. Favorable was defined the area supplied by the surgical bypass covered more than 2/3 of the middle cerebral artery (MCA) distribution; Moderate was defined that 1/3–2/3 of the MCA distribution was covered; and Poor was defined that only slight or no collateral circulation was observed.

### Evaluation of postoperative outcomes

The early postoperative complications and the long-term neurological function were used to evaluate the patients’ outcomes. We analyzed the early postoperative complications of patients, including cerebral infarction, cerebral hemorrhage, and cerebral hyperperfusion syndrome (CHS) within 7 days following revascularization. The diagnosis of postoperative CHS is based on the patient’s clinical manifestations, such as headache, altered consciousness, or symptoms of focal neurological deficit, combined with brain CT, MRI, T2WI, and FLAIR imaging to show cerebral edema in the surgical area [15]. Moreover, the study utilized the modified Rankin Scores (mRS) to assess the long-term neurological function of postoperative patients.

### Statistical analyses

Two-tailed Student’s *t*-tests were used when performed to compare the continuous variables of two groups, and the categorical variables were compared by the Pearson *χ*2 tests. The continuous variables between multiple groups were compared using the one-way ANOVA tests. Statistical analyses were conducted using SPSS v25 software (IBM, Armonk, USA). Two-sided values of *P* < 0.05 were considered statistically significant. All data generated and analyzed are available on reasonable request.

## Results

### Cohort characteristics

A total of 382 patients were reviewed in this study, of whom 278 were excluded, including 247 patients without preoperative or follow-up DSA and 8 patients who underwent direct bypass alone, while 23 patients underwent indirect bypass alone. Finally, a total of 104 patients with MMD who met the criteria were included in the study, 58 of them were ischemic MMD, 34 were hemorrhagic MMD, and 12 were others. Patients in this cohort ranged in age from 12 to 65 years, with 33 males and 71 females.

### Comparison between patients with or without ACA-PCA anastomoses

Patients were divided into PtoA group (66 patients) and non-PtoA group (38 patients) based on the presence of retrograde flow from the posterior circulation to the anterior circulation. Demographic and clinical characteristics did not differ significantly between the two groups. In addition, there was no statistical difference between the two groups in the postoperative collateral vessel establishment (*P* = 0.253) and the long-term neurological function at follow-up (*P* = 0.066) (Table 1).

**Table 1:** Characteristics between MMD patients with or without ACA-PCA anastomosis

	Non-PtoA group (38)	PtoA group (66)	*P* value
Age (years)	43.5 ± 11.2	42.7 ± 11.3	0.746
Gender (men/women)	15/23	18/48	0.198
BMI (kg/m^2^)	24.3 ± 3.4	24.3 ± 3.7	0.932
Smoking (%)	11 (28.9%)	9 (13.6%)	0.056
Alcohol (%)	9 (23.7%)	9 (13.6%)	0.333
Hypertension (%)	12 (31.6%)	22 (33.3%)	0.854
Diabetes mellitus (%)	5 (13.2%)	4 (6.1%)	0.215
Manifestation (%) Ischemia Hemorrhage Others			0.868
	20 (52.6%)	38 (57.6%)	
	13 (34.2%)	21 (31.8%)	
	5 (13.2%)	7 (10.6%)	
Time to onset (months)	6.3 ± 8.7	11.9 ± 17.2	0.063
Suzuki stage I II III IV V VI			0.545
	5	4	
	7	8	
	18	35	
	5	14	
	3	5	
	0	0	
Operation side (L/R)	20/18	32/34	0.684
PCA involvement	12 (31.6%)	18 (24.2%)	0.401
Follow-up duration (months)	13.3 ± 7.4	13.2 ± 11.3	0.940
Matsushima grade			0.253
Favorable	9 (23.7%)	14 (21.2%)	
Moderate	16 (42.1%)	38 (57.6%)	
Poor	13 (34.2%)	14 (21.2%)	
mRS at follow-up			0.066
0	23 (60.5%)	52 (78.8%)	
1	13 (34.2%)	13 (19.7%)	
2	2 (5.3%)	0 (0.0%)	
3	0 (0.0%)	1 (1.5%)	

*MMD* moyamoya disease, *BMI* body mass index, *ACA* anterior cerebral artery, *PCA* posterior cerebral artery, *mRS* modified Rankin Scale, *L* left, *R* right

### Comparison between patients with different grades of ACA-PCA anastomoses

The patients were scored in accordance with the different types and degrees of anastomoses between the ACA and PCA, of whom 38 received a score of 0 (non-PtoA group), 17 with a score of 1, 15 with a score of 2, and 34 with a score of 3. In terms of demographic and clinical characteristics, there were no significant differences between the groups of patients. Furthermore, the postoperative collateral vessel establishment (*P* = 0.243) and the long-term neurological function at follow-up were also not significantly different between the groups (*P* = 0.360) (Table 2).

**Table 2:** Characteristics between MMD patients with different grades of ACA-PCA anastomosis

	Grade 0 (38)	Grade 1 (17)	Grade 2 (15)	Grade 3 (34)	*P* value
Age (years)	43.5 ± 11.2	40.9 ± 13.5	41.9 ± 12.2	44.0 ± 9.8	0.780
Gender (men/women)	15/23	4/13	4/11	10/24	0.606
BMI (kg/m^2^)	24.3 ± 3.4	22.7 ± 3.3	24.8 ± 4.0	24.8 ± 3.7	0.224
Smoking (%)	11 (28.9%)	1 (5.9%)	0 (0.0%)	8 (23.5%)	0.041
Alcohol (%)	9 (23.7%)	3 (17.6%)	1 (6.7%)	5 (14.7%)	0.616
Hypertension (%)	12 (31.6%)	6 (35.3%)	5 (33.3%)	11 (32.4%)	0.994
Diabetes mellitus (%)	5 (13.2%)	1 (5.9%)	0 (0.0%)	3 (8.8%)	0.464
Manifestation (%)					0.826
Ischemia	20 (52.6%)	9 (52.9%)	10 (66.7%)	19 (55.9%)	
Hemorrhage	13 (34.2%)	5 (29.4%)	5 (33.3%)	11 (32.4%)	
Others	5 (13.2%)	3 (17.6%)	0 (0.0%)	4 (11.8%)	
Time to onset (months)	6.3 ± 8.7	9.4 ± 17.0	11.2 ± 16.0	13.5 ± 18.2	0.225
Suzuki stage I II III IV V VI					0.482
	5	2	2	0	
	7	2	2	4	
	18	7	6	22	
	5	3	4	7	
	3	3	1	1	
	0	0	0	0	
Operation side (L/R)	20/18	8/9	5/10	19/15	0.512
Follow-up duration (months)	13.3 ± 7.4	12.7 ± 7.4	11.0 ± 2.9	14.4 ± 14.8	0.733
Matsushima grade					0.243
Favorable	9	2	1	11	
Moderate	16	11	10	17	
Poor	13	4	4	6	
mRS at follow-up					0.360
0	23	12	12	28	
1	13	5	3	5	
2	2	0	0	0	
3	0	0	0	1	

*MMD* moyamoya disease, *BMI* body mass index, *ACA* anterior cerebral artery, *PCA* posterior cerebral artery, *mRS* modified Rankin Scale, *L* left, *R* right

### Patients with ACA-PCA anastomoses have higher rate of CHS

Furthermore, we compared the early complications of patients with and without ACA-PCA anastomoses and demonstrated that the incidence of CHS in the early period after revascularization in the non-PtoA group was significantly higher than that in the PtoA group (34.2% *vs* 16.7%, *P* = 0.041) (Table 3). Besides, there was no significant difference between the two groups in terms of other early postoperative complications, such as cerebral infarction and cerebral hemorrhage.

**Table 3:** Short-term complications between MMD patients with or without ACA-PCA anastomosis

	Non-PtoA group (38)	PtoA group (66)	*P* value
Cerebral hyperperfusion syndrome	13 (34.2%)	11 (16.7%)	0.041*
Cerebral infarction	1 (2.6%)	1 (1.5%)	0.690
Cerebral hemorrhage	0 (0.0%)	1 (1.5%)	0.446

*P < 0.05

## Discussion

MMD is a chronic cerebrovascular disease with the generation of collateral vessels [16–18]. There is reduced blood flow to the parts of the brain that are supplied by the MCA and ACA due to progressive stenosis of the internal carotid artery [19, 20]. As a result of the insufficient blood supply in the anterior circulation, small and fragile smoke-like vessels are formed at the skull base. In addition, compensatory blood flow from posterior to anterior circulation also increases through the vertebrobasilar artery, and collateral vessels from the external carotid artery (ECA) may anastomose with the intracranial vessels as well.

Currently, research on the pathophysiology of MMD has been relatively limited due to the rarity of its morbidity [21], while the majority have focused on the impact of different revascularization surgeries on the prognosis of MMD patients. Only a few studies have provided detailed descriptions of the different types of collateral circulation that naturally develop within MMD based on angiography results [9, 10, 20, 22–24]. In view of this, a number of questions remain in relation to MMD collateral circulation, including whether collateral circulation is associated with a particular type of pathology or stage of the disease or whether it can be used as a prognosis predictor.

Baltsavias et al. [20] were the first to provide a systematic and detailed description of the collateral circulation in MMD, including two superficial leptomeningeal anastomoses systems and two deep parenchymal anastomoses systems. Subsequently, Japanese scholars defined three types of anastomoses patterns based on the typical vessels responsible for hemorrhagic MMD, namely lenticulostriate anastomoses, thalamic anastomoses, and choroidal anastomoses [9]. Bonasia et al. [8] identified three major types of anastomoses that occur between the posterior and anterior circulation, including the collaterals from the posterior pericallosal artery, the collaterals from posterior choroidal arteries, and the pio-pial connections between PCA and ACA.

Among these collateral circulation systems, the most direct and effective way of compensation is the retrograde flow from the posterior circulation to the anterior circulation through existing collateral vessel pathways, such as the posterior pericallosal artery, thus replenishing the ischemic areas of the ACA and MCA. The posterior pericallosal artery is a branch of PCA, which belongs to the embryonic residual artery and plays an important role in the communication between anterior and posterior circulation in the embryonic period.

Our study demonstrated that the presence of ACA-PCA anastomoses did not significantly affect the outcome of postoperative collateral vessel establishment and long-term prognosis, regardless of the type or degree of anastomoses. Therefore, two hypotheses have been proposed to explain these observations. First, the establishment of collateral circulation following revascularization is primarily dependent on blood supply from the ECA, which is not closely related to the ACA-PCA anastomoses. It is important to note that retrograde flow supply from the posterior pericallosal artery mainly supplies deep brain areas, with relatively limited blood flow to the cortex. In addition, most of the cortical branches of the ACA-PCA anastomoses in the leptomeninges are located in the upper and posterior parietal lobes, covering a relatively small space within the MCA blood supply. As a result, ACA-PCA anastomoses play a limited role in establishing postoperative collaterals. Second, the growth of collateral vessels after revascularization surgery may be influenced by the individual’s level of angiogenic factors. According to recent research, platelet-derived growth factor (PDGF) and its receptor PDGFR are likely to play a key role in vessel formation in MMD [25, 26]. It was reported by Marushima et al. [27] that transfection of the PDGF-BB gene enhanced the formation of collateral vessels in mice following EMS. We can conduct a prospective study to validate this hypothesis by combining cerebral perfusion with angiogenic factor levels in cerebrospinal fluid in the future.

**Fig. 4:**
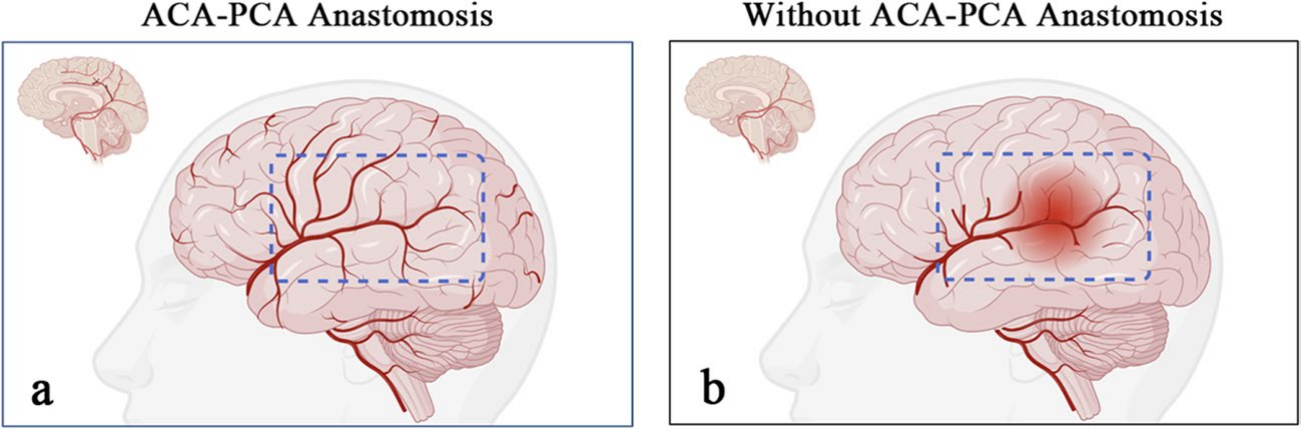
**a** Supposes that MMD patients with ACA-PCA anastomoses have more collateral vessels to accommodate the blood flow from the external carotid artery after revascularization, thereby preventing a rapid increase in cerebral blood flow and the development of cerebral hyperperfusion syndrome in the bypass area. On the other hand, the hypothesis of (**b**) is that patients without ACA-PCA anastomoses generally have fewer collateral vessels before surgery, and they are more likely to experience rapid increases in CBF after revascularization, which may lead to edema and cerebral hyperperfusion syndrome. Created with BioRender.com

Along with the long-term prognosis, we also examined the effect of the ACA-PCA anastomoses on early postoperative complications in patients with MMD. Our results showed that patients with ACA-PCA anastomoses had a significantly lower incidence of postoperative CHS than those without. There is a possibility that MMD patients with ACA-PCA anastomoses have more collateral vessels to accommodate the flow of blood from the ECA after revascularization, thereby preventing a rapid increase in CBF and the development of CHS in the bypass area (Fig. 4a). On the other hand, MMD patients without ACA-PCA anastomoses generally have fewer collateral vessels before surgery, and they are more likely to experience rapid increases in CBF after revascularization, which may lead to brain edema and CHS (Fig. 4b). Thus, we recommend that MMD patients without ACA-PCA anastomoses should be closely monitored following revascularization to avoid drastic blood pressure fluctuations and minimize postoperative complications. However, it should be noted that as patients receive a STA-MCA bypass, flow accommodation is primarily dependent on collateralization in the MCA territory; therefore, the presence of ACA-PCA anastomosis is considered an indirect indicator of general collateralization.

Study limitations also need to be considered. Due to the retrospective nature of this study, there may be some bias in the analysis of early complications and the assessment of long-term neurological function among patients with MMD. Additionally, the posterior pericallosal artery lies near the midline of the brain, so determining the side of the anastomoses based on anteroposterior images may result in errors. Third, the diagnosis of postoperative CHS is primarily based on the clinical manifestations of the patient and the corresponding radiographic findings, rather than on transcranial Doppler ultrasound and other methods of hemodynamic quantitative detection. Furthermore, the ACA-PCA anastomoses may also be compensated by the posterior communication artery as well as by anastomoses between the temporal branches of the PCA and the MCA. However, since these anastomoses are relatively rare, we did not include them in the study.

## Conclusion

In adult MMD patients, the preoperative ACA-PCA anastomoses may not have a significant impact on postoperative collateral vessel establishment and long-term neurological function, except for the early postoperative complication of CHS. Thus, patients without ACA-PCA anastomoses should be vigilant of the occurrence of CHS in the early stages after revascularization.

## Data Availability

All data generated or analyzed during this study are available from the corresponding authors on reasonable request.
